# Radical Resection for Locally Advanced Pancreatic Cancers in the Era of New Neoadjuvant Therapy—Arterial Resection, Arterial Divestment and Total Pancreatectomy

**DOI:** 10.3390/cancers13081818

**Published:** 2021-04-10

**Authors:** Yosuke Inoue, Atushi Oba, Yoshihiro Ono, Takafumi Sato, Hiromichi Ito, Yu Takahashi

**Affiliations:** Division of Hepatobiliary and Pancreatic Surgery, Cancer Institute Hospital, Japanese Foundation for Cancer Research, Tokyo 135-8550, Japan; atsushi.oba@jfcr.or.jp (A.O.); yoshihiro.ono@jfcr.or.jp (Y.O.); takafumi.sato@jfcr.or.jp (T.S.); hiromichi.ito@jfcr.or.jp (H.I.); yu.takahashi@jfcr.or.jp (Y.T.)

**Keywords:** pancreatic cancer, arterial resection, total pancreatectomy, neoadjuvant therapy

## Abstract

**Simple Summary:**

Aggressive arterial resection or total pancreatectomy in surgical treatment for locally advanced pancreatic cancer (LAPC) has gradually been encouraged thanks to new chemotherapy regimens such as FOLFIRINOX or Gemcitabine and nab-paclitaxel, which have provided more adequate patient selection and local tumor suppression, justifying aggressive local resection. The development of surgical techniques provides the safety of arterial resection (AR) for even major visceral arteries, such as the celiac axis or superior mesenteric artery. Total pancreatectomy has been re-evaluated as an effective option to balance both the local control and postoperative safety. In this review, we investigate the recent reports focusing on arterial resection and total pancreatectomy for locally advanced pancreatic cancer (LAPC) and discuss the rationale of such an aggressive approach in the treatment of PC.

**Abstract:**

Aggressive arterial resection (AR) or total pancreatectomy (TP) in surgical treatment for locally advanced pancreatic cancer (LAPC) had long been discouraged because of their high mortality rate and unsatisfactory long-term outcomes. Recently, new chemotherapy regimens such as FOLFIRINOX or Gemcitabine and nab-paclitaxel have provided more adequate patient selection and local tumor suppression, justifying aggressive local resection. In this review, we investigate the recent reports focusing on arterial resection and total pancreatectomy for LAPC and discuss the rationale of such an aggressive approach in the treatment of PC. AR for LAPCs is divided into three, according to the target vessel. The hepatic artery resection is the simplest one, and the reconstruction methods comprise end-to-end, graft or transposition, and no reconstruction. Celiac axis resection is mainly done with distal pancreatectomy, which allows collateral arterial supply to the liver via the pancreas head. Resection of the superior mesenteric artery is increasingly reported, though its rationale is still controversial. Total pancreatectomy has been re-evaluated as an effective option to balance both the local control and postoperative safety. In conclusion, more and more aggressive pancreatectomy has become justified by the principle of total neoadjuvant therapy. Further technical standardization and optimal neoadjuvant strategy are mandatory for the global dissemination of aggressive pancreatectomies.

## 1. Introduction

Pancreatic cancer (PC) is a dismal clinical entity [1]. For localized PCs, resection is the only chance for cure. Theoretically, R0 resection is one essential philosophy for cancer treatment even if the local tumor has invaded major visceral arteries. However, the aggressive biology of PC accompanied with occult metastasis has precluded simply extending the resection. Pancreatectomy is accompanied by high morbidity, and extended resection, including arterial resection (AR) or multi-organ resection, has been a challenge because of its substantial mortality [2,3]. Total pancreatectomy (TP) is an option to achieve R0 resection in locally advanced PCs. The rationale of TP for PC, however, has long been in controversy due to complicated short-time outcomes, including malnutrition or brittle diabetes, along with unsatisfactory long-term survival [4].

New-generation chemotherapies, i.e., FOLFIRINOX [5] or gemcitabine (GEM) + nab-paclitaxel (GNP) [6], have changed the paradigm of the treatment strategy for unresectable locally advanced (LA) PCs. In this review, we investigate the recent innovation of aggressive resection for LAPCs including AR or TP and discuss the future perspective of extended resections for advanced PCs.

## 2. Arterial Resections

### 2.1. Overview

Pancreatic ductal adenocarcinoma has an invasive nature, and our predecessor surgeons have tried to improve the prognosis by achieving R0 resection by extending resection. Fortner et al. reported the first series of extended resections named regional pancreatectomy [7]. This report described a novel approach of pancreatectomy for PCs, including TP and routine portomesentericosplenic confluence resection en bloc with the surrounding soft tissue. AR was concomitantly performed if needed. However, their results showed severe short-term outcomes and insufficient long-term survivals and was not accepted as a reasonable method to improve the treatment outcomes of LAPCs [2,7]. Since then, advances in surgical techniques and perioperative management have made venous resection and reconstruction during pancreatectomy safe [8,9]. Recent reports have documented favorable short-term outcomes of venous resection in patients with localized PCs [10,11]; however, the R0 resection rate, as well as long-term survival, remained unsatisfactory, because the most frequent site of cancer-positive margin was located at the superior mesenteric artery (SMA) margin [12,13], which could not be overcome by venous resection alone. Therefore, the necessity of more radical dissection, including arterial resection, remained and has become more prominent in the past two decades, although recent meta-analyses concluded that pancreatectomy with ARs remained a challenge, because it increased the complexity of the procedure and was associated with increased morbidity and mortality in comparison to non-AR pancreatectomies [14,15].

### 2.2. Management for the Involvement of the Superior Mesenteric Artery 

**Figure 1:**
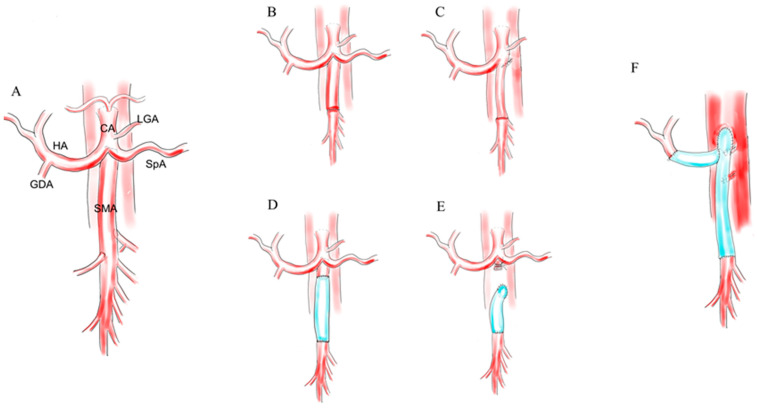
Reconstruction of the superior mesenteric artery. (**A**) Basic anatomy of relevant vessels in SMA resection. (**B**) Direct end-to-end anastomosis. (**C**) Transposition of SpA to be anastomosed with the distal stump of the SMA. (**D**) End-to-end anastomosis with graft interposition. (**E**) Graft interposition from the aorta to the distal stump of the SMA. (**F**) Combined resection and reconstruction of the HA and SMA using interposition grafts. HA, hepatic artery, SpA, splenic artery, GDA, gastroduodenal artery, SMA, superior mesenteric artery, MCA, middle colic artery and LGA, left gastric artery.

In advanced pancreatic uncinate cancers, the superior mesenteric artery (SMA) is the most common artery that is invaded and becomes a reason for unresectable or pathologically noncurative resection [12,13,16]. Until recent years, a large series of SMA resections for PCs was quite limited, and mortality after SMA resection had reportedly been higher than ordinal pancreatectomies, which discouraged the aggressive resection of LAPCs involving the SMA [17,18,19,20] (Table 1). As an alternative, periadventitial dissection (PAD) of the SMA had been proposed to pursue the local control of the peri-SMA region. Inoue et al. presented a standardized technique of SMA-PAD using the supracolic anterior artery-first approach, which resulted in no mortality over 158 patients, with a R0 rate of 74% [16,21]. Extended resection of the peri-SMA nerve plexus was assumed to cause neurogenic diarrhea, which would lead to insufficient patient recovery or adjuvant therapy. Inoue et al. documented that the incremental administration of an opium tincture according to the frequency of watery diarrhea was effective and easy to adjust to, with satisfactory diarrhea control, leading to sufficient adjuvant therapy introduction (83%) [16]. For more advanced tumors that cause encasement of the artery, SMA resection would be required. Recently, some high-volume centers with outstanding expertise in pancreatic resections have reported large series of arterial resections for PCs, including more than 30 cases of SMA resections [22,23]. Bachellier et al. [22] reported a large single-center series, including 34 SMA resections. They achieved the lowest mortality ever (5.1% of all patients with AR), which represented the improved safety of SMA resection and reconstruction. They mainly employed an end-to-end anastomosis using autografts such as a great saphenous vein and noted that reconstruction with an artificial graft caused thrombosis, leading to in-hospital mortality. Loos et al. [23] reported another large series involving 30 SMA resections with an acceptable mortality of 6.7%. They also performed a learning curve analysis and concluded that even an experienced pancreatic surgeon needed more than 12 cases of AR to minimize the mortality. An optimal reconstruction technique has never been established and likely depends on the length of a resected segment. Previous reports on SMA reconstruction employed end-to-end anastomosis or anastomosis to the aorta with or without graft interposition (Figure 1A,B,D,E) and a rotation of the splenic artery (SpA) (Figure 1C) [10,17,20,22,23,24,25,26,27,28,29,30,31,32,33,34]. Westermark et al. [35] proposed a safe technique of end-to-end anastomosis of the SMA. They recommended the Cattel-Braasch maneuver, wherein the total mesentery is detached from the retroperitoneum to facilitate a tension-free anastomosis. Sterile ice in a surgical towel was placed in the lower sub-mesocolic abdomen to reduce the warm ischemia of the small intestine. The Cattel-Braasch maneuver enabled tension-free anastomosis even after SMA resection of 4 cm in length. Accordingly, SMA resection is now no more an anecdotal tool but one possible option for LA pancreatic head cancers. Reports focusing on the long-term outcomes after SMA resection are still limited.

### 2.3. Resection of the Hepatic Artery 

**Figure 2:**
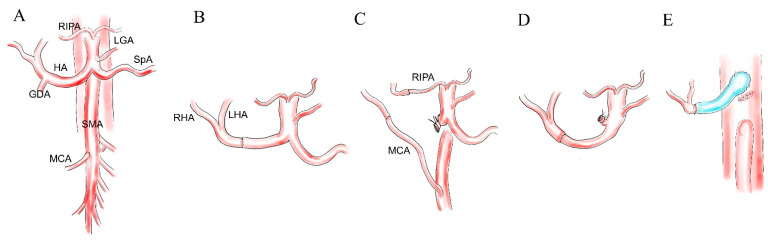
Reconstruction of the hepatic artery. (**A**) Basic anatomy of the relevant vessels in HA resection. (**B**) Direct end-to-end anastomosis. (**C**) Transposition of the MCA and RIPA to be anastomosed with the RHA and LHA. (**D**) Transposition of the SpA to be anastomosed with the proper HA. (**E**) Graft interposition from the aorta to the stump of the proper HA. HA, hepatic artery, RIPA, right inferior phrenic artery, SpA, splenic artery, GDA, gastroduodenal artery, SMA, superior mesenteric artery, MCA, middle colic artery, LGA left gastric artery, RHA, right hepatic artery and LHA, left hepatic artery.

Advanced cancers located at the pancreatic neck often invade the common and proper hepatic artery (HA), as well as the gastroduodenal artery (GDA). In such cases, segmental resection of the HA, including the root of the GDA, is suggested. If cancer invasion is limited and resected segment is short, end-to-end anastomosis is often possible. Recent guidelines have also described the combined HA or celiac axis (CA) resection as one of the putative options for LAPC [36]. Although a large series that specifically focuses on HA resection is limited, there are many small case series, including five to 20 patients who mainly underwent pancreaticoduodenectomy (PD) with concomitant resection of the HA until recently [10,17,20,24,25,26,27,29,30,31,32,37,38,39] (Table 2). Amano H et al. first reported a medium series of HA resections in which they described the details of techniques and outcomes about HA reconstruction. The in-hospital mortality rate was 7%, accompanied by an R0 rate of 80% and a median survival time (MST) of 12 months. The authors concluded that HA resection is justified only when surgery of R0 has taken place for selected patients with PC. Regarding the reconstruction technique of the HA, several reports described HA reconstruction, which was dominantly done by end-to-end anastomosis (Figure 2A,B) [24,25,26,27,30,31,32,37]. Short-segment resection of the HA was simple and safe and could be recommended as an entry procedure of AR for pancreatic surgeons who perform pancreatic head resection. In a case where the HA is resected in a long segment, arterial transposition (Figure 2C,D) [17,32] or interposition using the autograft to bridge between the celiac axis or aorta and proper HA is required (Figure 2E) [24,25,27,31]. To simplify and reduce the number of anastomoses, transposition of the SpA or colic artery should first be considered. The right inferior phrenic artery is an alternative option for a small orifice of the left HA. Although SpA transposition is usually performed with TP to gain enough length of the SpA pedicle, preservation of the pancreas tail would be possible if the left gastric artery (LGA) and great pancreatic artery are preserved. Desaki et al. reported a case series of SpA resection during PD mainly for PCs and documented that no clinically relevant splenic infarction was observed [40]. On the other hand, the omittance of HA reconstruction would be possible if we performed a specific preparation for HA resection. Miyazaki et al. proposed the novel management of HA resection with preoperative HA embolization to enhance the collateral hepatic arterial inflow [38]. After HA resection, backflow from the proper HA stump was observed. If the backflow was strong enough, they omitted HA reconstruction. In a 21-patient series, they reconstructed HA in only one patient, and eventually, 33% of the patients suffered postoperative liver infarction, but there was no in-hospital mortality.

### 2.4. Resection of the Celiac Axis 

CA resection for advanced pancreatic body cancer was an exceptional situation of arterial resection, wherein reconstruction of the hepatic artery was considered to be unnecessary because of the peripancreatic collateral arterial flow that originated from the SMA [41]. Pancreatic body cancers frequently involve the celiac–hepatic artery system, and distal pancreatectomy with celiac axis resection (DP-CAR) was a reasonable choice to achieve an en-bloc eradication of the tumor and its invasion. The concept of DP-CAR was a modification of the Appleby procedure originally for advanced gastric cancers [41]. The first report about DP-CAR was written by Hishinuma et al. in 1991, and they documented the preservation of the whole stomach during CAR and distinguished DP-CAR from the Appleby procedure in that the stomach was preserved [42]. Afterward, several small series of DP-CARs were reported [43,44,45,46,47], and in 2007, Hirano et al. first described the short- and long-term outcomes of the standardized DP-CAR [48]. They reported 23 patients who underwent DP-CARs with no mortality and had acceptable overall survival (five-year survival rate, 42% and median survival time, 21 months). This pivotal report encouraged pancreatic surgeons worldwide to perform DP-CAR as a promising option to balance surgical and oncological safety. However, as the cases accumulated, ischemic complications involving the stomach or liver became prominent, as well as post-pancreatectomy hemorrhage, caused by the insufficient drainage of postoperative pancreatic fistula, leading to non-negligible mortality [49,50,51,52,53,54] (Table 3). Ischemic gastropathy or stomach perforation were complications specific to DP-CARs, which often included resection of the LGA, as well as the left gastroepiploic artery. Moreover, radical retroperitoneal dissection during DP-CAR includes resection of the left inferior phrenic artery. These sacrifices of critical gastric inflows potentially lead to life-threatening gastropathy [55]. As for liver infarction, collateral hepatic flow via the GDA was theoretically sufficient for liver perfusion. However, excessive dissection of the GDA sometimes leads to arterial stenosis, which causes depression of the hepatic arterial flow [56]. Depression of the proper hepatic artery induces recurrent cholangitis, liver abscess or cholecystitis. Cholecystitis was reported to be one possible cause of postoperative major intervention [50,55]. Therefore, the gallbladder should be resected routinely during DP-CAR. In the early years, preoperative arterial embolization of the HA or LGA to enhance the collateral flow was encouraged to avoid ischemic complications. However, recent reports found no positive impact of arterial embolization on the prevention of postoperative ischemic complications [55,56,57,58]. Another possible resolution is an intraoperative reconstruction of the LGA. Sato et al. first described reconstruction of the LGA to avoid ischemic gastropathy after DP-CAR [59]. The authors used a pedicle of the middle colic artery as an origin of the arterial supply. The right branch of the middle colic artery is usually away from the pancreatic body cancer and used as a suitable counterpart of the LGA. The efficacy of the anastomosis should be confirmed promptly and objectively after anastomosis. Oba et al. reported the intraoperative evaluation of the patency of LGA anastomosis using indocyanine green fluorescence imaging [60]. By these managements, the safety of DP-CARs would be improved.

## 3. Total Pancreatectomy 

TP was reported by Rockey et al. for the first time [61]. Although TP was attempted to improve the survival of patients with PC with the rationale to avoid anastomosis-related morbidity and mortality in early years [62,63], Warren et al. documented that TP led to pancreatic endocrine and exocrine insufficiency, resulting in brittle diabetes due to a lack of endocrine and malabsorption caused by exocrine deficiency [64]. Later, TP was indicated with the intention to improve the local control in extensive pancreatic cancers [7]. However, as was described by Fortner et al., a simple extension of resection resulted in poor short-term outcomes accompanied by unsatisfactory survival [2]. In the 1980s, TP was attempted to eradicate multicentric carcinogenesis in the whole pancreas, but it failed to improve the survival of patients with PS, because the incidence of tumor multicentricity proved to be low [4,65]. Therefore, TP has been discouraged for the curative treatment of PCs [66]. After 2000, the introduction of long-acting insulin formulations facilitated the easy control of blood sugar levels after TP. As a result, endocrine-related mortality has been rarely reported ever since. As for exocrine insufficiency, diarrhea was the most frequent sequelae after TP, and 23.5% of patients who underwent TP still had symptoms, despite pancreatic enzyme administration [67]. Moreover, malabsorption causes postoperative steatohepatitis, which potentially leads to life-threatening hepatic decompensation [68]. Hata et al. identified female gender, malnutrition and insufficient pancreatic enzyme substitution as significant prognostic factors of post-TP steatohepatitis and suggested that high-dose pancreatic enzyme replacement therapy might have preventive effects on hepatic steatosis occurring after a pancreatectomy [69]. Anyway, the development and standardization of the surgical technique fostered by the case accumulation and centralization of complicated procedures has gradually made the surgical outcomes of TP an acceptable level, like partial pancreatectomy [70,71,72,73]. Long-term survivals have gradually become better and better. Until the middle of the 2000s, the MST of patients who underwent TP for PCs was about one year or less [4,74,75]. Schmidt et al. reported a substantial improvement in survival after TP for pancreatic neck cancers, documenting an MST of 18 months [76]. After 2010, a large series comprising 289 patients with TP for PCs documented an MST of 18.1 months [72]. Accordingly, TP was gradually reappraised as a reasonable option to achieve a cure for selective patients with PC [70,72,77,78,79,80,81,82,83] (Table 4). If TP was applied to LAPCs to obtain a cure or long-term survival, we would have to consider the quality of life after TP, as well as the absolute surgical safety or survival time. Recently, several reports documented a significant reduction of physical functioning [84] or both the physical and emotional composite scores [85,86]. Stoop et al. stated in the latest report that the quality of life after TP was reduced in comparison to the general population but remained stable compared with the preoperative situation [84]. They demonstrated the challenges of endocrine (96% of patients involved) and exocrine insufficiency (64% of patients involved) after TP and claimed that the management of both insufficiencies should be improved further to overcome the quality of life reduction after TP.

## 4. Recent Evolution of Radical Pancreatectomies in the Era of New Regimens and Future Perspective

### 4.1. Recent Reports of Extremely Radical Pancreatectomy

The respective techniques of arterial resection or total pancreatectomy have gradually matured and become common among experienced pancreatic surgeons; however, extremely radical pancreatectomy involving major arterial resection with or without total pancreatectomy is still controversial in that long-term survival is not considered worth carrying the surgical risks for patients with LAPC [3,17,70,71,72]. However, the introduction of new-generation chemotherapy regimens such as FOLFIRINOX [5] or GNP [6] has gradually changed the paradigm of indication for these surgical challenges. In recent years, multiple high-volume pancreatic centers have reported extremely radical pancreatectomy after intensive neoadjuvant therapy (NAT) using FOLFIRINOX or GNP.

Tee et al. first reported a large series of AR combined with new-generation NAT for advanced PCs [33]. In this study, 111 patients underwent pancreatectomy with AR, including any hepatic (54%), any celiac (44%), any superior mesenteric (14%) or multiple ARs (14%), with revascularization in 55% (Figure 1F). TP was performed on 20 (18%) patients. The majority of cases underwent planned AR (77%), and most of the procedures were performed post-2010 (78%). The most common indication for pancreatectomy was for PC in 87 (78%) patients. Of these patients, 65 (75%) were treated with neoadjuvant systemic chemotherapy that included FOLFIRINOX, GNP or both, with the majority (88%) also receiving sequential chemoradiation with a total dose of 50.4Gy with various radiation sensitizers. Ninety-day major morbidity (≥grade III) and mortality was 54% and 13% mainly due to post-pancreatectomy hemorrhage, postoperative pancreatic fistula or ischemia. They emphasized that a significant decrease in mortality was achieved in patients who underwent ARs post-2010 (9% compared with 29% in patients before 2010, *p* = 0.02). From the same group, Truty et al. reported a systematic classification of CAR, which included three levels according to the extent of the resection: class 1, celiac only, class 2, celiac and PHA and class 3, SMA additional to class 1 or 2 [57]. Ninety-day mortality was 10%, with a significant improvement in the last 50 consecutive cases (4%). The R0 resection rate (88%) was associated with chemoradiation (*p* = 0.004). The MST was 36.2 months, superior from the neoadjuvant chemotherapy (8.0 vs. 43.5 months). Truty et al. also reported a large series comprising 194 borderline resectable or LAPC [87]. En-bloc venous and/or arterial resection was required in 125 (65%) patients, with 94% of patients achieving R0 margins. TP was performed in 25 (13%) patients. The 90-day mortality was 6.7%. Among patients without mortality, epochally favorable survival outcomes were obtained (the median, one-year, two-year and three-year overall survivival (OS) rates were 58.8 months, 96%, 78% and 62%, respectively). They emphasized the efficacy of total neoadjuvant therapy (TNT) with favorable prognostic factors: extended duration (six cycles) of neoadjuvant chemotherapy, optimal post-chemotherapy CA19-9 response and major pathological response. Bachellier et al. reported a large AR series for PCs with excellent postoperative outcomes [22]. The most impactful point was that this study included 35 SMA resections, which was the largest ever. The overall mortality and morbidity were 5.1% and 41.5%, respectively. Preoperative radiation was not employed, assumably to secure the safety of complicated AR of the major visceral arteries. TP was performed in 18 (15%) patients. Some patients (75.4%) underwent NAT. The median, one-year, three-year and five-year OS rates after resection were 13.7 months, 59%, 13% and 12%, respectively. They identified that R0 resection (hazard ratio: 0.60, *p* = 0.01) and pathological venous invasion (hazard ratio: 1.67, *p* = 0.04) were independent prognostic factors. Loos et al. reported the largest AR series (195 patients) for LAPCs recently [23]. They compared AR with periadventitial dissection (PAD; *n* = 190), which was an optional technique to achieve R0 resection in borderline resectable or LAPCs, and revealed higher rates of postoperative pancreatic fistula (4.2% after PAD vs. 10.3% after AR; *p* = 0.022), post-pancreatectomy hemorrhage (4.7% vs. 14.9%; *p* = 0.001), ischemia (4.2% vs. 15.9%; *p* < 0.0001) and relaparotomy (12.6% vs. 26.9%; *p* = 0.001) after AR. The overall mortality rate of AR was higher than that of PAD (12.8% vs. 4.7%; *p* = 0.005). Although the mortality rate became lower and lower through the study period, AR remained more dangerous than PAD. The authors concluded even experienced pancreatic surgeons needed a learning curve of 15 ARs to safely perform the procedure. These results indicated the difficulty of AR to be disseminated globally. The median and five-year OS rates were 21.5 months and 15%, respectively, after PAD and 17.7 months and 9% after AR (*p* = 0.099). These results were attributed to more advanced stages and less incidences of NAT in the AR group.

### 4.2. En-Bloc Arterial Resection or Arterial Divestment?

There still remains controversy over the issue of whether we choose AR for major vessels or not, especially for the SMA. Even in highly selected patients, the SMA resection is regarded as difficult to be generalized. To balance surgical and oncological safety, the arterial divestment technique has been proposed as an alternative for SMA resection. “Divestment” means “undressing” or “circumferential dissection”. The detailed technique and outcomes of arterial divestment were described in recent reports from the Heidelberg group [88,89]. The SMA was dissected using an artery-first approach through a wide Kocher maneuver, and if needed, a Cattel-Braasch maneuver was added. The authors recommend intraoperative sampling of the periadventitial tissue around the SMA, and if the cancer was positive, divestment was first attempted. Cai et al. recommended in their report that peri-adventitial dissection should be done with cold dissection using the tip of a right-angled clamp or the nonworking tip of energy devices [89]. Burn injury on the arterial wall would be a risk of postoperative aneurysm. If the dissection was difficult due to direct encasement, finally, AR was employed. To select among the three choices: divestment, AR or aborting resection before the point of no return, an artery-first approach is mandatory. The safety of the divestment technique was reported by a recent article from the same group of Heidelberg [23]. Inoue et al. [16] described the details of periadventitial dissection around the SMA, which resulted in no mortality by the use of an artery-first approach. It did not preclude postoperative recovery or adjuvant therapy if the neurogenic diarrhea was adequately controlled. However, the safe utilization of this technique has never been generalized. Sabater et al. [90] conducted the first randomized trial to compare the oncological and surgical outcomes between artery-first PD and standard PD. The authors concluded that they found no difference either in the R0 resection rates (67.9 % vs. 77.3 %, *p* = 0.194) or in the postoperative complications (overall morbidity rate; 67.9% vs. 73.3%, *p* = 0.484) in patients undergoing artery-first PD versus standard PD. Although this trial included only resectable PCs and other periampullary malignancies, and their conclusions could not be applied directly to the management of LAPCs, this technique should be carefully applied by an expert pancreatic surgeon at a high-volume center. Another important matter is when and how we decide the approach to the SMA. Habib et al. [91] also encouraged SMA divestment for selected patients after new-generation NAT. They also indicated the usefulness of the preoperative radiological finding of circumferential SMA encasement. Halo sign, wherein the SMA was surrounded by hypodense tissue without narrowing, was potentially a candidate for resection using arterial divestment. On the other hand, string sign, wherein the SMA was surrounded by periadventitial tissue forming an irregular narrowing (like a string), was not a candidate for R0 resection, even with arterial divestment. Habib et al. and the John’s Hopkins group did not regard a patient with string sign as an adequate candidate for resection, because they could not justify SMA resection due to the high morbidity and mortality. However, the radiological change after NAT did not represent a pathological regression of the tumor cells, and decision-making by the preoperative findings alone would include the risk of overdiagnosis and loss of chance for a cure. Del Chiaro also advocated intraoperative decision-making of the divestment or AR [92]. The author also recommended performing the divestment technique by the surgical team experienced in AR, because we have to prepare for unexpected arterial injury during SMA dissection, which requires complex vascular reconstruction.

On the other hand, Truty et al. [87] strongly recommended a planned en-bloc resection, even for the SMA. Their recent report still included a high mortality rate (9 out of 71 LAPC patients) after aggressive AR, but they stated that the safety of AR has become more robust recently and documented a surprisingly high R0 rate and long-term survival. Actually, the intraoperative judgement of periadventitial cancer invasion requires a test dissection, which potentially cuts into the cancer tissue. The superiority of planned en-bloc portal vein resection in obtaining R0 to unplanned venous resection after a test dissection was recently documented [93]. The en-bloc approach is exactly the principle of regional pancreatectomy suggested by Fortner et al. [7], and the reappraisal of regional en-bloc resection has been reported, such as for portal vein resection [94]. If the safety of AR is guaranteed, the same theory should be justified in SMA resection as well. For pancreatectomy with complicated AR, the efficacy of concomitant TP has been reappraised. The total removal of the pancreatic gland makes the procedure safer by eliminating the problem of pancreas fistula and its potentially fatal effect on arterial anastomosis [95,96]. This strategy, which was originally suggested at the dawn of the radical resection of PCs, has become justified after the improvement of the perioperative management of TP patients through several decades.

### 4.3. Rationale of Total Neoadjuvant Therapy

Another recent topic relevant to extremely radical pancreatectomy for PCs is the rationale of TNT. TNT has been advocated for LA gastrointestinal cancers, i.e., esophageal cancers [97] or rectal cancers [98,99], wherein the surgical burden of resection likely hampers prompt postoperative recovery and adequate adjuvant systemic chemotherapy. For LAPCs, due to a lack of effective regimens, TNT has long been out of the question, and the efficacy of TNT was suggested only recently. Murphy et al. reported a prospective single-arm phase II trial evaluating the efficacy of TNT using FOLFIRINOX for LAPCs with the primary endpoint of the R0 resection rate [100]. This report was the first concrete evidence of TNT for PCs. Forty-nine LAPC patients were enrolled. Eight cycles of FOLFIRINOX were administered, followed by short- or long-course chemoradiotherapy, depending on the radiological findings after FOLFIRINOX. Thirty-nine (80%) patients completed eight cycles. One patient (2%) had a radiographic complete response. Twenty-three patients (49%) had a partial response, while 21 (45%) had a stable disease. Two patients (4%) had a progressive disease by the response evaluation criteria in solid tumours (RECIST) criteria. Thirty-four patients (69%) underwent surgical resection. Finally, 30 (61%) patients achieved R0 resection. TNT with FOLFIRINOX was feasible and provided a favorable long-term survival (median progression-free survival was 17.5 months (95% CI: 13.9–22.7), and median MST was 31.4 months (95% CI, 18.1–38.5)). For LAPCs, intensive neoadjuvant therapy has already become a consensus, and the next issue is how we can standardize the optimal contents, dose and duration of NAT. Moreover, scientifically reliable evidence for neoadjuvant therapy for PCs [101,102] is still sparse compared to adjuvant therapy [103,104,105,106] so far. Whether or not we should really omit adjuvant therapy remains unclear.

## 5. Conclusions

In this review, the recent development of radical pancreatectomy, including arterial resection, arterial divestment or total pancreatectomy, was discussed. Thanks to the recent improvement of chemotherapy using multiple agents, both tumor suppression and patient selection have become pragmatic. Simple resection of the HA or CA and TP has likely become a matured technique. To implement ERP including SMA resection or combined major arterial resections, the further accumulation of cases, the establishment of a standardized technique and optimal neoadjuvant therapy should be pursued.

## Figures and Tables

**Table 1:** Previous reports about resection of the superior mesenteric artery.

Author	Year	Country	*N*	NAT (%)	Procedures	Reconstruction Method	Study Period	Mortality (%)
Li [24]	2004	China	11	ND	PD	Graft 8, interposition from the aorta 3	1994–2003	ND
Nakao [10]	2006	Japan	3	ND	PD, TP	ND	1981–2005	35.7 **
Yekebas [20]	2008	Germany	3	ND	PD, DP	EEA 1, graft 2	1994–2005	33
Amano [17]	2009	Japan	12	13 **	PD, TP	EEA 3, SpA transposition 7 *, graft 2	2005–2009	17
Boggi [25]	2009	Italy	6	ND	PD	EEA 1, graft 5	1987–2004	4.0 **
Martin [26]	2009	USA	2	100	PD, TP	Graft 2	1999–2007	0
Kitagawa [19]	2011	Japan	17	ND	PD	EEA 1, graft 16	2002–2011	12
Bockhorn [27]	2011	Germany	3	ND	PD, TP	Graft 3	1994–2004	14 **
Rehders [28]	2012	Germany	4	ND	PD	EEA 3, graft 1	2004–2010	ND
Gong [29]	2013	China	10	ND	PD	ND	2006–2011	6.7 **
Sgroi [30]	2015	USA	4	38 **	PD	EEA 4	2003–2013	ND
Glebova [31]	2016	USA	2	28 **	PD	EEA 1, graft 1	1989–2014	ND
Perinel [32]	2016	France	6	67	TP	SpA transposition 6	2008–2014	0
Tee [33]	2018	USA	15	75 **	PD, DP, TP	EEA, graft, or reconstruction ^†^	1990–2017	7.0
Loveday [34]	2019	Canada	10	94 **	PD, DP, TP	EEA, interposition from the aorta ^†^	2009–2016	3.2 **
Bachellier [22]	2020	France	34	75 **	PD, DP, TP	EEA or graft 34 ^†^	1990–2017	5.7
Loos [23]	2020	Germany	30	49 **	PD, DP, TP	EEA, graft, transposition ^†^	2003–2019	6.7

NAT, neoadjuvant therapy, ND, not described, PD, pancreaticoduodenectomy, TP, total pancreatectomy, DP, distal pancreatectomy, EEA, end-to-end anastomosis and SpA, splenic artery. * The hepatic artery was anastomosed to the SpA with total pancreatectomy; ** Incidence among all patients with arterial resection. ^†^ Each number was not documented.

**Table 2:** Previous reports about resection of the hepatic artery.

Author	Year	Country	*N*	NAT (%)	Procedures	Reconstruction Method	Study Period	Mortality (%)
Li [24]	2004	China	8	ND	PD	EEA 5, graft 3	1994–2003	ND
Nakao [10]	2006	Japan	9	ND	PD, TP	ND	1981–2005	ND
Yekebas [20]	2008	Germany	10	ND	PD, TP, DP	EEA 10	1994–2005	0
Amano H [17]	2009	Japan	15	13 ^†^	PD, TP	EEA 3, GDA 4 *, SpA 6 **, Others 3	2005–2009	6.7
Boggi [25]	2009	Italy	12	ND	PD	EEA 6, graft 5, no reconstruction 1	1987–2004	4 ^†^
Martin [26]	2009	USA	3	33	PD, TP	EEA 3	1999–2007	0
Bockhorn [27]	2011	Germany	18	ND	PD, TP	EEA 10, graft 8	1994–2004	14 ^†^
Gong [29]	2013	China	5	ND	PD	ND	2006–2011	6.7 ^†^
Amano R [37]	2015	Japan	7	100	PD, TP	EEA 6, no reconstruction 1	2012–2013	0
Sgroi [30]	2015	USA	7	38 ^†^	PD	EEA 7	2003–2013	ND
Glebova [31]	2016	USA	18	28 ^†^	PD	EEA 15, graft 2, no reconstruction 1	1989–2014	ND
Perinel [32]	2016	France	6	0	TP	SpA 3, no reconstruction 3 ^‡^	2008–2014	0
Miyazaki [38]	2017	Japan	21	43	PD, TP	EEA1, no reconstruction 20	2019–2015	0
Tee [33]	2018	USA	60	75 ^†^	PD, DP, TP	EEA or graft or reconstruction ^§^	1990–2017	13
Loveday [34]	2019	Canada	10	94 ^†^	PD, DP, TP	EEA, interposition from the aorta ^†^	2009–2016	3.2 ^†^
Bachellier [22]	2020	France	29	75 ^†^	PD, DP, TP	EEA or graft 20 ^§^, no reconstruction 9 ^§^	1990–2017	5.1 ^†^
Loos [23]	2020	Germany	85	49 ^†^	PD, DP, TP	EEA, graft, transposition ^§^	2003–2019	16.7

EEA, end-to-end anastomosis, ND, not described, PD, pancreaticoduodenectomy, TP, total pancreatectomy, DP, distal pancreatectomy, GDA, gastroduodenal artery and SpA, splenic artery. * The replaced hepatic artery was anastomosed to the GDA. ** The hepatic artery was anastomosed to the SpA with total pancreatectomy. ^†^ Incidences among all patients with arterial resection. ^‡^ Includes patients who had replaced HA. ^§^ Each number was not documented.

**Table 3:** Previous reports of distal pancreatectomy with celiac axis resections (DP-CARs).

Author	Year	Country	*N*	Study Period	Preoperative Embolization (%)	LGA Flow Preservation (%)	Ischemic Complication (%)		Mortality (%)
							Stomach	Liver	
Klompmaker [49]	2019	Europa	191	2000–2016	38	12	11	23	9.5
Nakamura [50]	2016	Japan	80	1998–2015	100	6.3	29	6	5
Yamamoto [53]	2017	Japan	72	2001–2011	ND	ND	ND	ND	4.2
Okada [55]	2018	Japan	50	2004–2017	92	46	10	56	8
Yoshitomi [58]	2019	Japan	38	2010–2016	74	0	10	3	3
Ocuin [48]	2016	USA	30	2007–2015	ND	0	7	ND	14
Yoshiya [49]	2019	Japan	20	2008–2018	80	0	0	ND	0
Beane [51]	2015	USA	20	2011–2012	ND	0	0	0	10
Oba [57]	2019	Japan	18	2014–2017	0	89	11	ND	0

LGA, left gastric artery and ND, not described.

**Table 4:** Previous reports about total pancreatectomy for pancreatic cancers.

Author	Year	Country	*N*	Study Period	Mortality (%)	R0 Resection Rate (%)	Median Survival Time (Months)
Brooks [74]	1989	USA	48	1970–1986	8.3	ND	12
Launois [75]	1993	France	47	1968–1986	15	ND	8
Karpoff [4]	2001	USA	35	1983–1998	5.7	82	7.9
Schmit [76]	2007	USA	33	1992–2006	6	100	18
Reddy [70]	2009	USA	100	1970–2007	8	78	12
Nathan [77]	2009	USA	376	1998–2004	8.6	ND	15
Hartwig [71]	2015	Germany	289	2001–2012	7.8	ND	18
Satoi [78]	2016	Japan	45	2001–2011	0	76	17
Johnston [79]	2016	USA	2582	1998–2004	5.5	76	15
Xiong [80]	2017	China	50	2009–2015	6	90	18
Passeri [81]	2019	USA	807	1998–2006	5.6	76	17
Hashimoto [82]	2020	Japan	1393	2013–2016	1.1	ND	ND
Stoop [83]	2020	Sweden	90	2008–2017	3.4	ND	ND

ND, not described.
